# Supplementary material for: The Surgical Imprint: How Operative Trauma May Shape Radiation Tolerance After Prostatectomy
Source: Cancers (Basel). 2025 Aug 18;17(16):2685. doi: 10.3390/cancers17162685 (PMC12385124; doi:10.3390/cancers17162685)

SUPPLEMENTARY MATERIALS

Table S1: The surgical "imprint": how operative trauma pre-conditions the radiation micro-environment

**Dominant feature of open prostatectomy**	**Immediate tissue effect**	**Down-stream radiosensitizing consequence**
Wide fascial dissection and direct muscle incision	Capillary rupture, hematomas	Persistent hypoxia → impaired DNA-repair, greater fixation of radiation-induced double-strand breaks
Prolonged operative time / retraction	Ischemia–reperfusion burst	Surge in reactive oxygen species (ROS) primes endothelium for micro-vascular apoptosis during RT
Extensive lymph-venous transection	Pelvic oedema, slow drainage	Delayed clearance of cytokines → prolonged inflammatory milieu that augments rectal/bladder mucosal damage
Higher systemic cytokine surge (IL-6, TNF-α, TGF-β1)	Acute and chronic inflammation	TGF-β-driven myofibroblast activation → early fibro-remodeling stiffens bladder/rectal walls, raising point doses for any given PTV margin

SUPPLEMENTARY MATERIALS

Figure S1: proposed mechanistic pathway linking surgical technique to enhanced radiation toxicity.
(The surgical approach, open versus minimally invasive, establishes distinct biological conditions in the postoperative prostate bed. Open surgery induces greater early tissue disruption (fibrosis, hypoxia, lymphatic damage), which in turn alters the microenvironment through endothelial dysfunction and inflammatory cytokine release. These factors collectively increase tissue susceptibility to radiation injury, resulting in higher rates of acute gastrointestinal and genitourinary toxicity following salvage radiotherapy.)

SUPPLEMENTARY MATERIALS

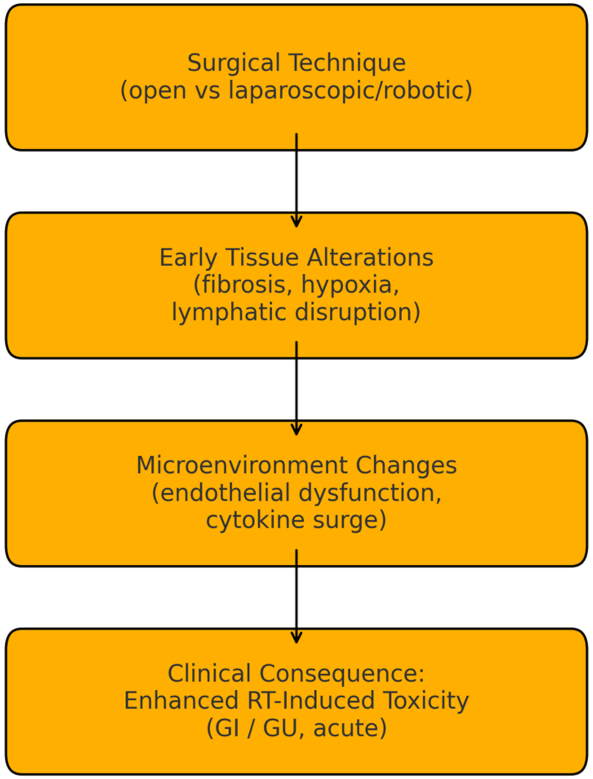

Section 2.2 Overview

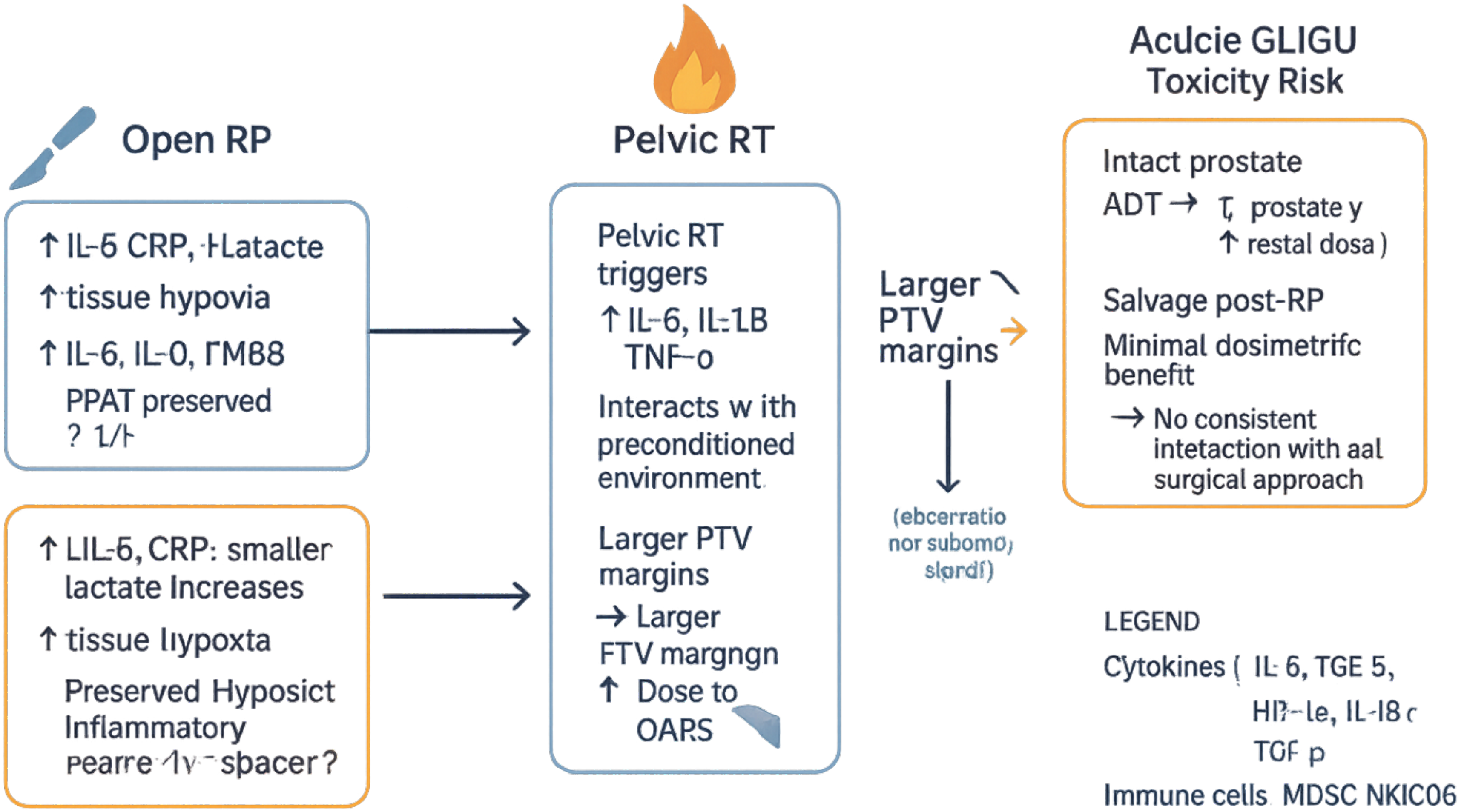

Supplement: Supplementary file 1 [file cancers-17-02685-s001.zip › cancers-3776179-supplementary.pdf]
